# Selective Activation of p120^ctn^-Kaiso Signaling to Unlock Contact Inhibition of ARPE-19 Cells without Epithelial-Mesenchymal Transition

**DOI:** 10.1371/journal.pone.0036864

**Published:** 2012-05-09

**Authors:** Hung-Chi Chen, Ying-Ting Zhu, Szu-Yu Chen, Scheffer C. G. Tseng

**Affiliations:** 1 Tissue Tech, Inc., Ocular Surface Center, and Ocular Surface Research & Education Foundation, Miami, Florida, United States of America; 2 Department of Ophthalmology, Chang Gung Memorial Hospital, Linkou, Taiwan; 3 Graduate Institute of Clinical Medical Sciences, Chang Gung University College of Medicine, Taoyuan, Taiwan; University of California, Merced, United States of America

## Abstract

Contact-inhibition ubiquitously exists in non-transformed cells and explains the poor regenerative capacity of *in vivo* human retinal pigment epithelial cells (RPE) during aging, injury and diseases. RPE injury or degeneration may unlock mitotic block mediated by contact inhibition but may also promote epithelial-mesenchymal transition (EMT) contributing to retinal blindness. Herein, we confirmed that EMT ensued in post-confluent ARPE-19 cells when contact inhibition was disrupted with EGTA followed by addition of EGF and FGF-2 because of activation of canonical Wnt and Smad/ZEB signaling. In contrast, knockdown of p120-catenin (p120) unlocked such mitotic block by activating p120/Kaiso, but not activating canonical Wnt and Smad/ZEB signaling, thus avoiding EMT. Nuclear BrdU labeling was correlated with nuclear release of Kaiso through p120 nuclear translocation, which was associated with activation of RhoA-ROCK signaling, destabilization of microtubules. Prolonged p120 siRNA knockdown followed by withdrawal further expanded RPE into more compact monolayers with a normal phenotype and a higher density. This new strategy based on selective activation of p120/Kaiso but not Wnt/β-catenin signaling obviates the need of using single cells and the risk of EMT, and may be deployed to engineer surgical grafts containing RPE and other tissues.

## Introduction

Dysfunction and death of retinal pigment epithelium (RPE) constitute the final common pathway in age-related macular degeneration, the leading cause of legal blindness among the elderly (reviewed by Binder et al. [1]), as well as retinitis pigmentosa [2] and Stargardt's macular dystrophy [3]. As a monolayer of cuboidal cells, the RPE monolayer rests on the Bruch's membrane and plays a pivotal role in maintaining photoreceptor functions *in vivo* (reviewed by Strauss [4]) The RPE monolayer is also terminally differentiated and mitotically quiescent *in vivo* presumably due to contact inhibition [1]. Recently, cell-based therapy has emerged as a promising strategy for treating diseases characterized by RPE dysfunction [5], leading to an increasing need of engineering RPE in an *ex vivo* environment. Hence, it is also important to develop a new strategy of switching proliferation in contact-inhibited RPE without losing its normal phenotype to epithelial-mesenchymal transition (EMT).

EMT is a common cell biological process prevalent in embryogenesis but becomes pathogenic in a post-natal life because it leads to the loss of epithelial cell characteristics and the gain of mesenchymal phenotype, including expression of α-smooth muscle actin (α-SMA) (reviewed by Kalluri et al. [6]). Experimentally, EMT is commonly noted in pre-confluent RPE [7], [8] in porcine RPE explants when cell junctions are disrupted by EGTA followed by addition of TGF-β2 [9]. EMT also occurs in immediately confluent ARPE-19 cells in response to epidermal growth factor (EGF) [10], TGF-β [10]–[12] or in combination [10] without knowing whether these confluent ARPE-19 cells have ceased proliferation due to contact inhibition. EMT is associated with overexpression of ZONAB or knockdown of ZO-1 that is used to increase mouse RPE proliferation [13]. Clinically, EMT occurs in a number of pathological diseases involving RPE (reviewed by Saika et al. [14]), such as proliferative vitreoretinopathy, in which RPE undergoes EMT to become fibroblastic and contractile cells, leading to tractional retinal detachment and blindness (reviewed by Nagasaki et al. [15] and Pastor et al. [16]). Importantly, the detrimental outcome of proliferative vitreoretinopathy is also attributed to proliferation of abnormal RPE [15]. It remains unclear whether EMT is inevitable upon loss of contact inhibition or whether different growth factors might have different impacts on proliferation, even if EMT is elicited.

Intercellular junctions include gap junctions, adherent junctions (AJs), and tight junctions, among which AJs play an important role in controlling many cellular behaviors, including proliferation, differentiation, and survival (reviewed by Perez-Moreno et al. [17]). Although not fully elucidated, the mechanism governing contact inhibition-mediated mitotic block likely involves signaling transmitted from AJs to the nucleus (reviewed by Jamora et al. [18], Perez-Moreno et al. [17], and Matter et al. [19]). Conceivably, two signaling pathways could be elicited via β-catenin and p120-catenin (hereafter p120), respectively, when AJs are disrupted. The former is known as the canonical Wnt pathway, in which β-catenin, if stabilized in the cytoplasm without ubiquitin-mediated degradation, can be translocated into the nucleus where it acts as a transcriptional co-activator through binding with T cell factor/lymphoid enhancer factor (TCF/LEF) transcription factors (reviewed by Willert et al. [20] and Nelson et al. [21]). The latter may trigger the p120/Kaiso pathway, in which nuclear translocated p120 relieves the repressor activity of Kaiso, a member of BTB/POZ-ZF transcription factor family [22]-[24] (also reviewed by Daniel [25]). It has been known that p120 negatively regulates RhoA and other Rho GTPases [26], [27]. However, it is unclear whether and how activation of RhoA following the release of p120 inhibition can be linked to p120 nuclear trafficking and subsequent signaling.

To address the aforementioned issues, we adopted the model of post-confluent contact-inhibited ARPE-19 cells and noted that disruption of contact inhibition by EGTA followed by addition of EGF and FGF-2 indeed triggers EMT by activating canonical Wnt signaling, which is also sufficient in activating Smad/ZEB1/2 signaling resulting in EMT. Moreover, we discovered a novel strategy of unlocking such mitotic block by p120 siRNA knockdown, which selectively activates p120/Kaiso and RhoA-ROCK, but does not affect Wnt/β-catenin signaling. Consequently, a more compact RPE monolayer ensues and retains a normal phenotype without EMT after prolonged transfection of p120 siRNA for 15 days followed by withdrawal. These results highlight the feasibility of deploying this novel strategy of selective activation of p120/Kaiso signaling to engineer RPE and other similar tissues without the use of single cells and to switch proliferation on in contact-inhibited tissues without risking EMT.

## Results

### Disruption of contact inhibition by EGTA followed by EGF+FGF-2 leads to proliferation with EMT

**Figure 1:**
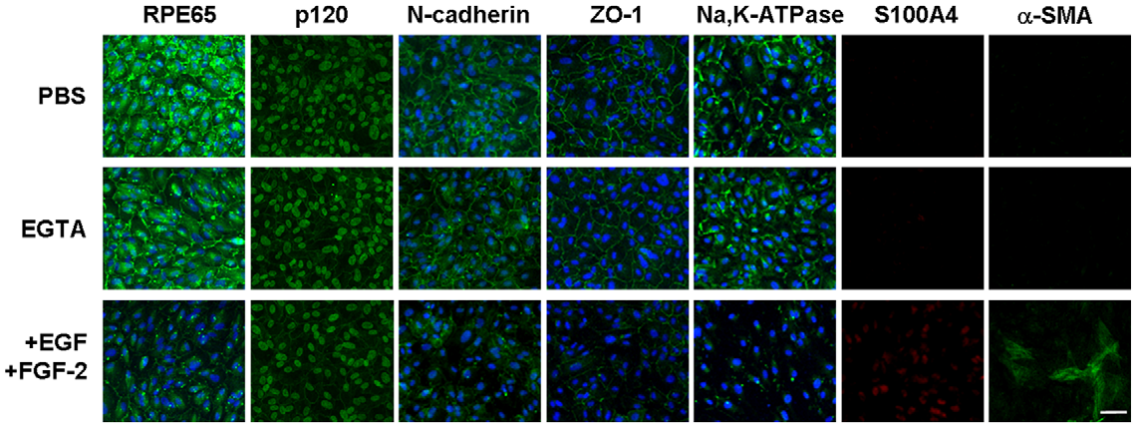
Contact inhibition disrupted by EGTA followed by EGF+FGF-2 leads to EMT. ARPE-19 cells cultured to day 7 post-confluence were treated with PBS, 1 mM EGTA, or 1 mM EGTA plus 10 ng/ml EGF and 20 ng/ml FGF-2 for 1 day. Immunostaining to RPE65, N-cadherin, ZO-1, and Na, K-ATPase was detected in the intercellular junction in PBS and EGTA, but was notably diminished in EGTA+EGF+FGF-2. Positive staining to S100A4 and α-SMA emerged. Scale bar indicates 100 μm.

In the model of ARPE-19 cells, our preliminary experiments showed that BrdU labeling was still detectable 4 days and became non-detectable 7 days after confluence (Fig. S1). Hence, we chose to conduct all subsequent experiments at day 7 post-confluence. We also noted that disruption of cell junctions by EGTA was critical to unlock contact inhibition as no BrdU labeling was noted when EGF and/or FGF-2 was added if EGTA was not also added (not shown). In contrast, with EGTA, additional EGF+FGF-2 caused a significant increase of BrdU labeling index to 21 %. Nonetheless, such a treatment also altered expression pattern of several known RPE markers including RPE65, a key enzyme within the visual cycle and a differentiation marker of RPE cells located in cytoplasm and at membrane [4] and of ARPE-19 cell line [28], [29], Na,K-ATPase [30], N-cadherin [31], [32] and ZO-1 [33]. As compared to their predominant cytolocalization at intercellular junctions in the control treated with PBS, EGTA alone did not cause any significant alteration (Fig. 1). However, EGTA+EGF+FGF-2 abrogated their staining in the intercellular junctions (Fig. 1). To determine if the loss of RPE phenotype was coupled with EMT, we studied the expression S100A4 and α-SMA as a gain of a mesenchymal phenotype (reviewed by Zeisberg et al. [34] and Schneider et al. [35]). Our result showed that α-SMA was detected only after EGF+FGF-2 were added (Fig. 1). These results collectively indicated that contact inhibition unlocked by EGTA with EGF+FGF-2 was accompanied by loss of the normal RPE phenotype and gain of a mesenchymal phenotype suggestive of EMT.

### Contact inhibition is uniquely unlocked by down-regulation of p120 without EMT

**Figure 2:**
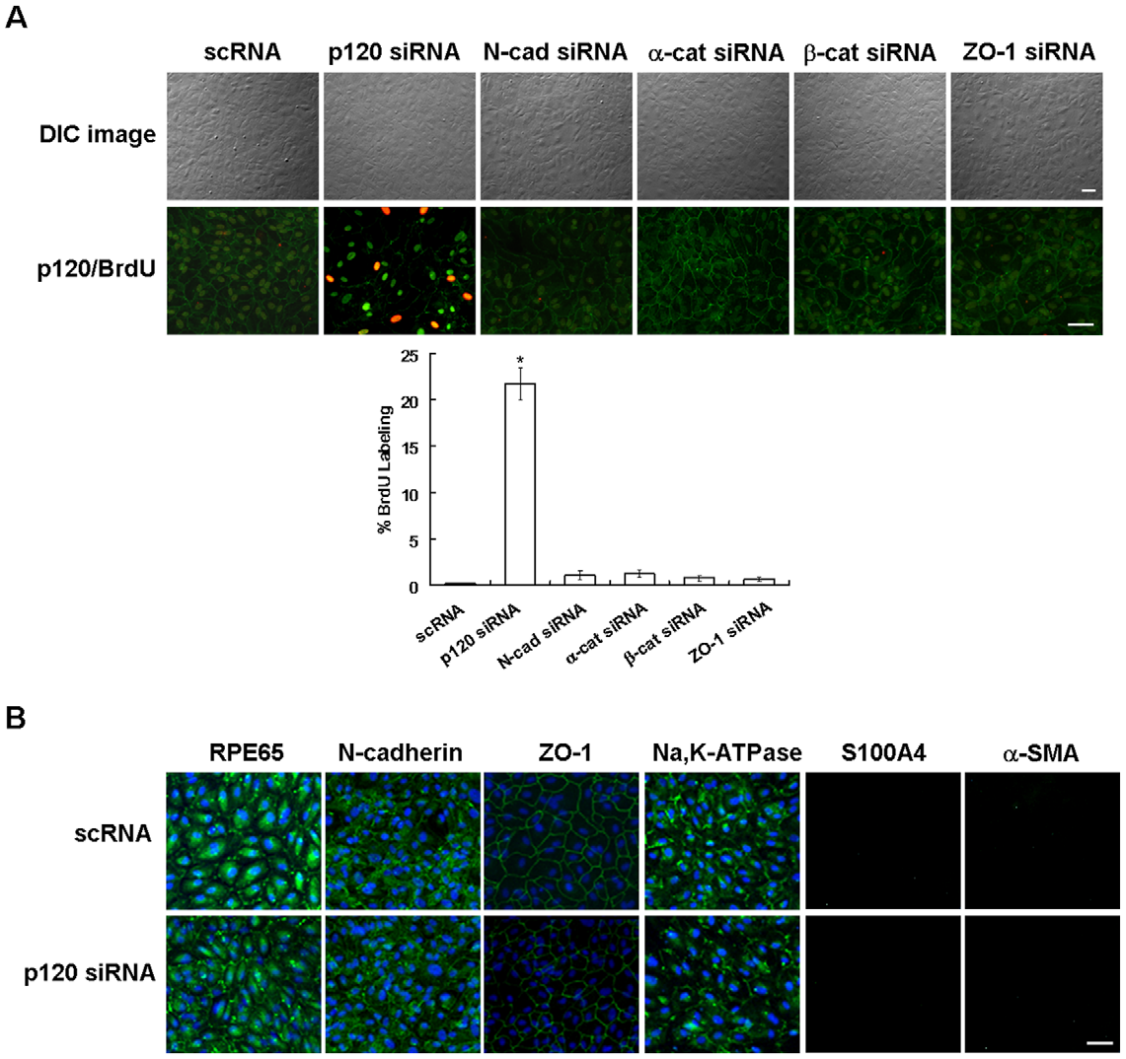
Contact inhibition is unlocked without EMT by p120 knockdown. ARPE-19 cells cultured to post-confluence day 7 were transfected with 100 nM scRNA or siRNAs to such junctional components as p120, N-cadherin, α-catenin, β-catenin, and ZO-1 for 2 days, and cultured in the presence of 10 μm BrdU for 4 h before fixation in acid/methanol. (A) DIC cell morphology was not altered by any siRNA compared to the scRNA control. BrdU nuclear labeling was only increased in cells transfected with p120 siRNA, where some were colocalized with nuclear translocation of p120. (B) Immunostaining of RPE65, N-cadherin, ZO-1, Na,K-ATPase, S100A4, and α-SMA in cells transfected with p120 siRNA was similar to that of the scRNA control. Scale bar indicates 100 μm and * denotes P<0.05.

Because disruption of cell junctions by EGTA+EGF+FGF-2 ran the risk of EMT (Fig. 1), we adopted a different approach by siRNA to perturb components of intercellular junctions including p120, N-cadherin, α-catenin, β-catenin, or ZO-1. The knockdown efficiency of these siRNAs was verified by both qRT-PCR and Western blotting (Fig. S2). For p120, we have tested the efficiency of 4 different siRNAs, single or in combination, and noted that the chosen p120 siRNA exhibited the best knockdown efficiency, i.e., ∼95% at the mRNA level and ∼80% at protein level. Furthermore, the efficiency was not improved by various combinations. Differential Interference Contrast (DIC) microscopy showed that the cell morphology was not altered by any siRNA compared to the scRNA control (Fig. 2A). BrdU nuclear labeling was only increased in cells transfected with p120 siRNA, which was partially colocalized with increased nuclear staining of p120 (Fig. 2A, n = 6, *P*<0.05). When compared to the scRNA control, the membranous and cytoplasmic staining of RPE65, intercellular junctional staining pattern of N-cadherin, ZO-1, and membranous staining of Na,K-ATPase remained unchanged in p120 siRNA-transfected cells (Fig. 2B). Unlike EGTA+EGF+FGF-2 group, cells transfected with p120 siRNA did not express cytoplasmic S100A4 and α-SMA (Fig. 2B). These results collectively indicated that the mitotic block could also be unlocked by p120 siRNA knockdown without perturbing the normal RPE phenotype and without running the risk of developing EMT.

### Wnt and Smad/ZEB signalings are activated by EGTA+EGF+FGF-2 but not by p120 knockdown

**Figure 3 pone-0036864-g003:**
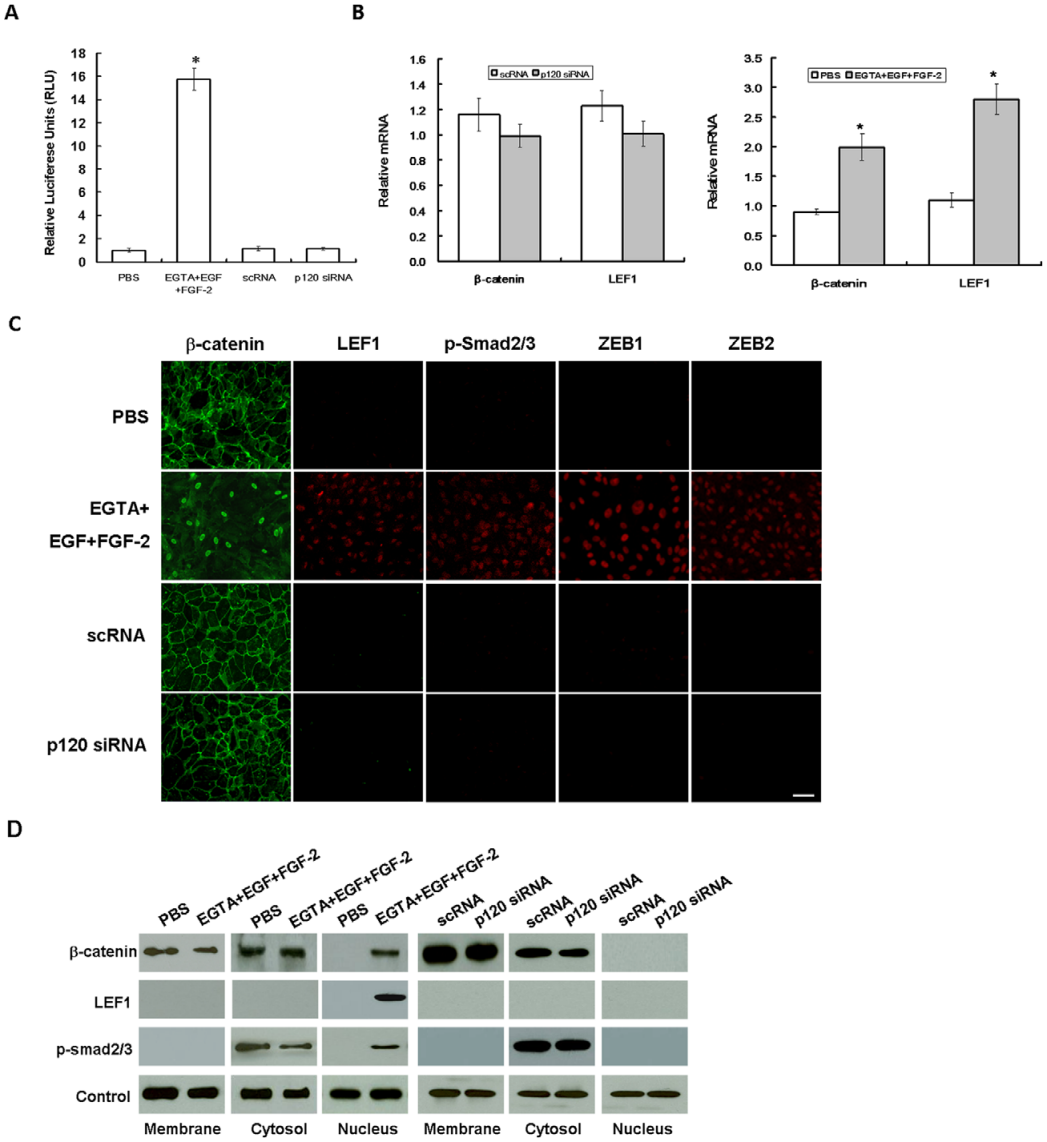
Activation of Wnt and Smad/ZEB signaling by EGTA+EGF+FGF-1 but not p120 knockdown. ARPE-19 cells cultured to day 7 post-confluence were treated with PBS or 1 mM EGTA plus 10 ng/ml EGF and 20 ng/ml FGF-2 for 1 day with or without 5 ng/ml XAV939, or transfected with 100 nM scRNA or p120 siRNA for 2 days. (A) The TCF/LEF1 promoter activity was silent in PBS, scRNA, or p120 siRNA, dramatically elevated to nearly 16-fold by EGTA+EGF+FGF-2 (P<0.05), but completely abolished by XAV939 (not shown). (B) qRT-PCR showed that β-catenin and LEF1 transcripts were up-regulated 2.2- and 2.5-fold, respectively (P<0.05), in EGTA+EGF+FGF-2, but remained unchanged in p120 siRNA (P>0.05). (C) Immunostaining reveal increased nuclear staining of β-catenin, LEF1, p-Smad2/3, ZEB1, and ZEB2 in EGTA+EGF+FGF-2 but not p120 siRNA. Scale bar indicates 100 μm. (D) Western blot analysis confirmed that the β-catenin level decreased in the membranous extract but increased in the nuclear extract, while the LEF1 level increased in the nuclear extract in EGTA+EGF+FGF-2, while there was no such change by p120 siRNA. Connexin43, α-tubulin, and histone serve as the loading control for membranous, cytosolic, and nuclear extracts, respectively.

Because S100A4 is a transcriptional target of canonical Wnt signaling [36], we suspected that the EGTA+EGF+FGF-2 treatment might have activated the Wnt signaling. We thus transfected post-confluent ARPE-19 cells with a plasmid containing TCF/LEF promoter construct and simultaneously treated the cells with XAV939, a specific Wnt inhibitor, which promotes phosphorylation-dependent degradation of β-catenin by increasing the activity of the destruction complex [37]. The results showed that the TCF/LEF promoter activity was low in cells treated with PBS but elevated 15-fold in cells treated with EGTA+EGF+FGF-2, and such an elevated activity was abolished by XAV939 (manuscript submitted). In contrast, the activity remained unchanged in cells transfected with scRNA or p120 siRNA (Fig. 3A, n = 3, *P*<0.05). qPCR disclosed 2.3- to 2.5-fold elevated levels of β-catenin and LEF1 mRNAs in cells treated with EGTA+EGF+FGF-2 but not p120 siRNA (Fig. 3B, n = 3, *P*<0.05). Immunostaining also confirmed that β-catenin was decreased in the cell junctions but increased in the nuclei, while there was also a notable increase of LEF1 staining in the nuclei of cells treated with EGTA+EGF+FGF-2 but not p120 siRNA (Fig. 3C). Western blot further confirmed a 2-fold reduced level of junctional β-catenin but a dramatic increase of nuclear levels of β-catenin and LEF1 proteins in cells treated with EGTA+EGF+FGF-2 but not with p120 siRNA (Fig. 3D). These results indicated that canonical Wnt signaling was activated by EGTA+EGF+FGF-2 but not by p120 siRNA.

A number of studies have shown that EMT is induced in a Smad-dependent manner (reviewed by Xu et al. [38]) via direct interaction of nuclear p-Smad2/3 with the zinc finger factors ZEB1 and ZEB2 [39], [40]. Immunostaining indeed confirmed that nuclear staining of p-Smad2/3, ZEB1, and ZEB2 was only noted in cells treated with EGTA+EGF+FGF-2 but not p120 siRNA (Fig. 3C). Western blot further confirmed a 2-fold decrease of the cytoplasmic level but a dramatic increase of the nuclear level of p-Smad2/3 in cells treated with EGTA+EGF+FGF-2 but not with p120 siRNA (Fig. 3D). These results suggested that the mitotic block unlocked by EGTA+EGF+FGF-2, but not p120 siRNA, was associated with activation of canonical Wnt and Smad/ZEB signaling.

### BrdU labeling is increased by nuclear release of Kaiso only following nuclear translocation of p120

**Figure 4 pone-0036864-g004:**
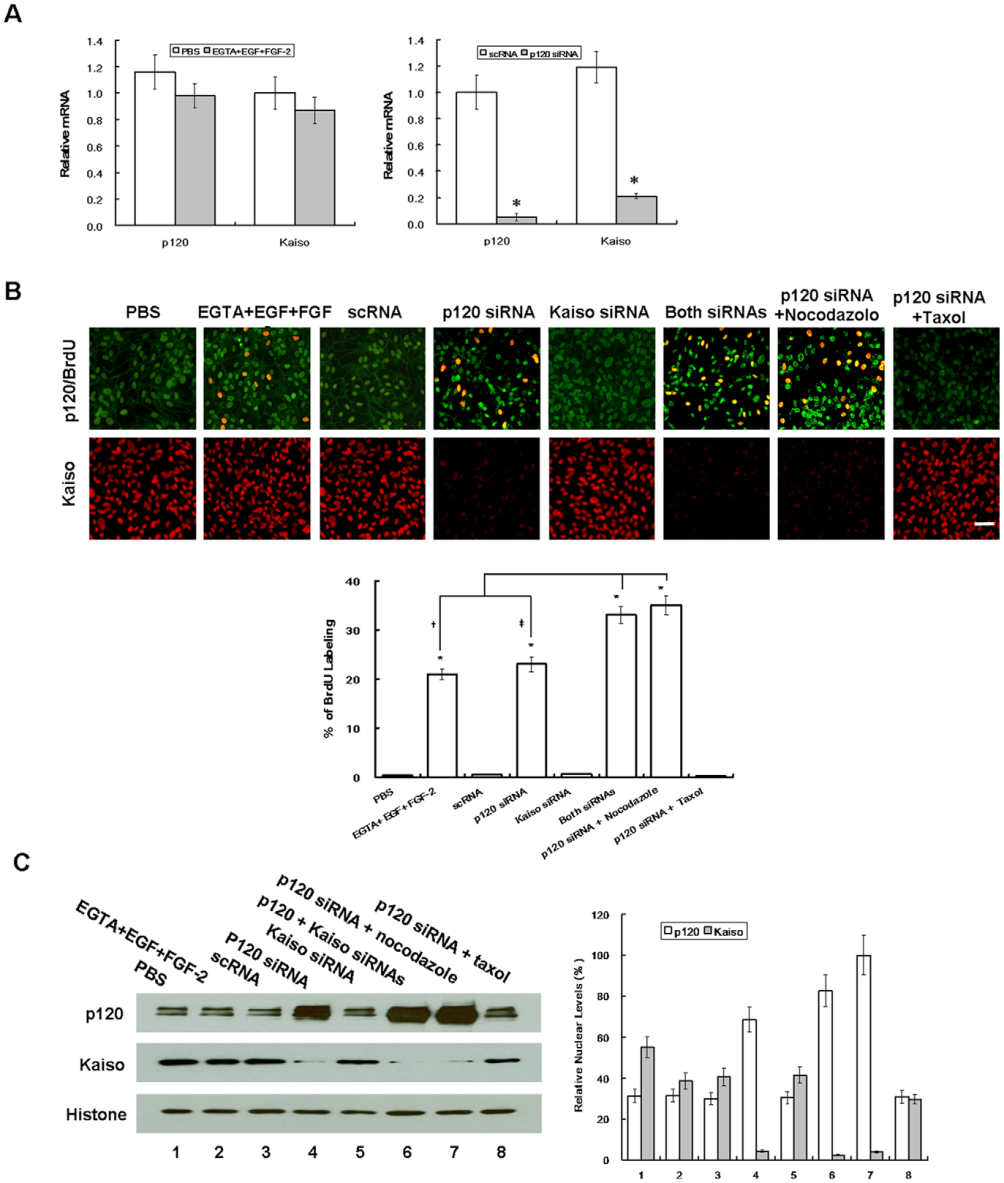
Correlation between BrdU labeling and nuclear translocation of p120 and nuclear release of Kaiso. ARPE-19 cells cultured to day 7 post-confluence were treated with PBS or 1 mM EGTA with 10 ng/ml EGF plus 20 ng/ml FGF-2 for 1 day, or transfected with 100 nM scRNA or siRNA to p120, Kaiso, or both for 2 days, and cultured in the presence of 10 μm BrdU for 4 h before fixation with acid/methanol. During p120 siRNA transfection, some cultures were added with 5 μg/ml nocodazole or 10 ng/ml taxol. (A) qRT-PCR showed that p120 and Kaiso transcripts remained unchanged by EGTA+EGF+FGF-2 but were 20- and 6-fold suppressed, respectively, by p120 siRNA. (B) As expected, the BrdU labeling index was significantly increased by EGTA+EGF+FGF-2 and p120 siRNA. Compared to scRNA, the BrdU labeling was not changed by Kaiso siRNA alone, but was significatly incrased when Kaiso siRNA was added with p120 siRNA. Compared to p120 siRNA, the BrdU labeling was further promoted from 23% to 35% by nocodazole, but decreased to 0.2% by taxol. Such changes of BrdU labeling index was correlated with increasing p120 nuclear staining and decreasing Kaiso nuclear staining. (C) The above changes were supported by Western blot which showed an inverse relationship between the p120 level and the Kaiso level in nuclear extracts using histone as the loading control. Scale bar indicates 100 μm, while *, †, and ‡ denote *P*<0.05.

Although p120 is usually found at cell junctions and undergoes nucleocytoplasmic shuttling [23], [41], [42], controversy of nuclear p120 in normal or tumor cells exists [25]. We suspected that such a controversy might stem from the difference in the fixative used for immunostaining. In fact, the nuclear p120 staining was not apparent when RPE monolayers were treated with 4% paraformaldehyde that has been used by others [42]–[44] but became apparent when fixed by 25% acetic acid plus 75% methanol (Fig. S3). To determine whether nuclear p120 triggered by p120 siRNA (Fig. 2A) was responsible for the release of nuclear Kaiso's transcriptional repression as suggested [25], we first examined the expression of p120 and Kaiso transcripts by qPCR. Compared to the control scRNA group, cells transfected with p120 siRNA expressed 20- and 6-fold less of p120 and Kaiso transcripts, respectively (Fig. 4A, n = 3, *P*<0.05). To confirm the above results, we performed knockdown of 3 other p120 siRNAs with target sequences: 5′GCCAGAGGTGGTTCGGATA3′ [43], 5′AACGAGGTTATCGCTGAGAAC3′ [45], and 5′GCGATTGCTTCGAAAGGCTCGTGAT3′ (designed by us), and noted similar results (not shown). As a contrast, EGTA+EGF+FGF-2 treatment did not alter the level of p120 and Kaiso transcripts. As expected, the BrdU labeling index was promoted by both EGTA+EGF+FGF-2 and p120 siRNA (Fig. 4B, n = 6, *P*<0.05). p120 siRNA knockdown markedly reduced p120 staining in the cell junctions but increased that in the nucleus, where it was partially colocalized with increased nuclear BrdU staining and with reduced nuclear Kaiso staining. Because Kaiso transcript was also lowered by p120 siRNA (Fig. 4A), we would like to rule out whether the use of Kaiso siRNA, which decreased Kaiso transcript and protein, but to a less extent (Fig. S2A, B), could have indirectly contributed the aforementioned effect by p120 siRNA. Our finding showed that Kaiso siRNA alone did not promote the BrdU labeling and p120 nuclear staining, nor did it decrease nuclear Kaiso staining. In contrast, simultaneous Kaiso siRNA and p120 siRNA knockdown synergistically promoted the BrdU labeling and p120 nuclear staining as well as decreased Kaiso nuclear staining (Fig. 4B). The aforementioned immunostaining differences of p120 and Kaiso were confirmed by quantitation of both protein levels by Western blotting using nuclear extracts (Fig. 4C, n = 3, *P*<0.05). We found that there was a strong inverse correlation between the p120 nuclear level and the Kaiso nuclear level, suggesting that p120 nuclear translocation dictated Kaiso nuclear release in promoting BrdU labeling.

To further prove that nuclear translocation of p120 was crucial for Kaiso nuclear release, we examined the effect of nocodazole and taxol, which depolymerizes and stabilizes the microtubule network, respectively, to affect the p120 cytosolic pool, thus indirectly affecting p120 nuclear translocation [46]. Our result showed that nocodazole increased the nuclear p120 staining from 3- (samples treated with p120 siRNA) to 6-fold (samples treated with p120 siRNA+nocodazole), while taxol reduced nuclear p120 to the control level (Fig. 4C). Consequently, the nuclear Kaiso level was decreased 2-fold by nocodazole but increased to the control level by taxol. The aforementioned changes in nuclear p120 and Kaiso levels were also reflected by the extent of nuclear p120 and Kaiso staining and the BrdU labeling. Collectively, these results further supported that the mitotic block unlocked by p120 siRNA knockdown was mediated by p120/Kaiso signaling and that nuclear translocation of p120 played a pivotal role in releasing the nuclear Kaiso level to unlock the mitotic block in post-confluent ARPE monolayers.

### Nuclear p120-mediated proliferation is controlled by RhoA-ROCK signaling

**Figure 5:**
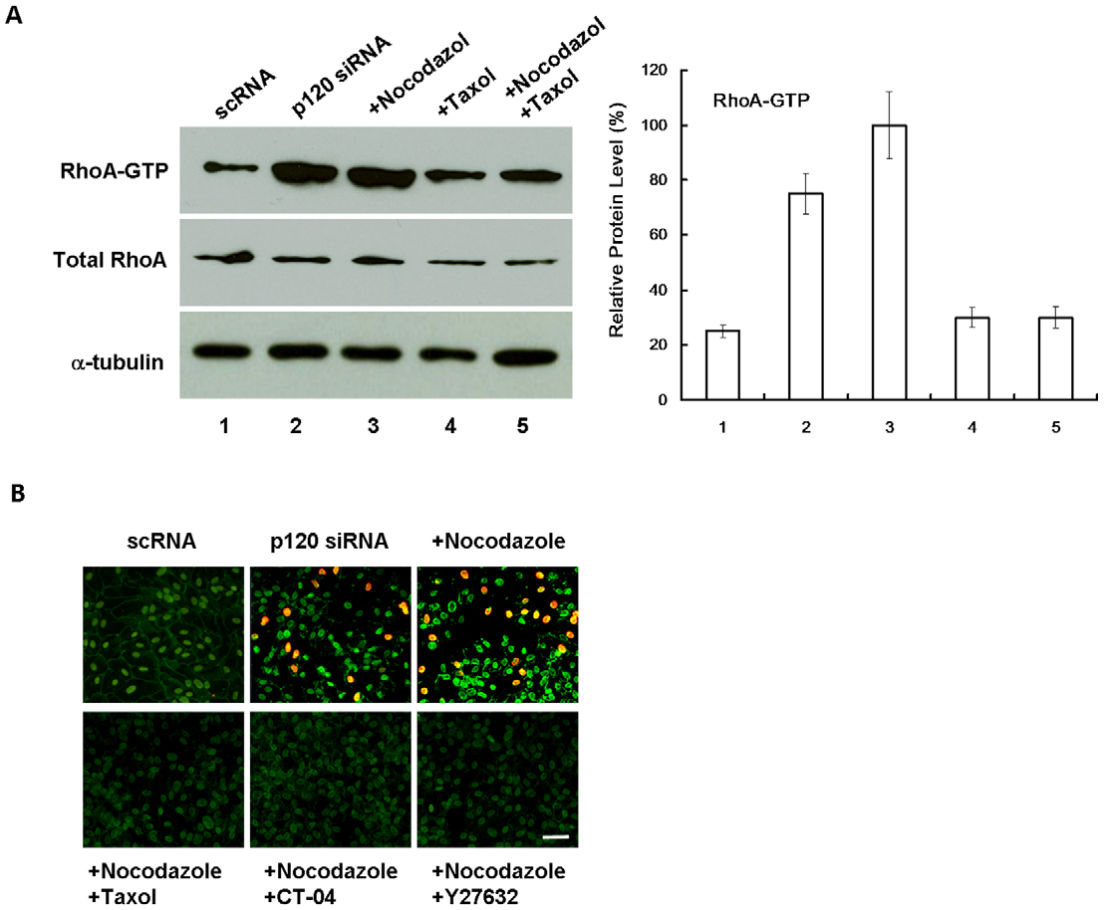
RhoA-ROCK signaling controls nuclear p120 translocation and BrdU labeling. (A) The level of active RhoA was promoted 5-fold by p120 siRNA and 7-fold with addition of nocodazole. The level of active RhoA promoted by p120 siRNA or p120 siRNA+nocodazole was inhibited by taxol to the baseline. (B) Nuclear BrdU labeling and p120 staining promoted by p120 siRNA+nocodazole was abolished by Taxol, CT-04 (RhoA inhibitor), or Y27632 (ROCK inhibitor). Scale bar indicates 100 μm.

p120 is known to interact and stabilize microtubules [47] and to inhibit RhoA and other Rho GTPases [26], [27]. Activation of RhoA signaling can destabilize microtubule [48]. Because destabilization of microtubules by nocodazole facilitated p120/Kaiso signaling (Fig. 4), we wondered whether RhoA was activated by p120 siRNA in a similar manner to nocodazole resulting in proliferation mediated by p120 nuclear translocation. Indeed we noted that the level of active RhoA was increased 5-fold by p120 knockdown and 7-fold with additional nocodazole (Fig. 5A). In contrast, the level of active RhoA promoted by p120 siRNA or p120 siRNA plus nocodazole was suppressed by Taxol to the baseline level. Similarly, nuclear p120 staining and BrdU labeling promoted by p120 siRNA plus nocodazole was abolished by taxol, CT-04 (a RhoA inhibitor), or Y27632 (a ROCK inhibitor) (Fig. 5B). These results suggested that activation of RhoA-ROCK signaling was correlated with destabilization of microtubules, p120 nuclear translocation, and BrdU labeling.

### Prolonged p120 knockdown followed by withdrawal leads to a more compact monolayer with normal ARPE phenotype without EMT

**Figure 6:**
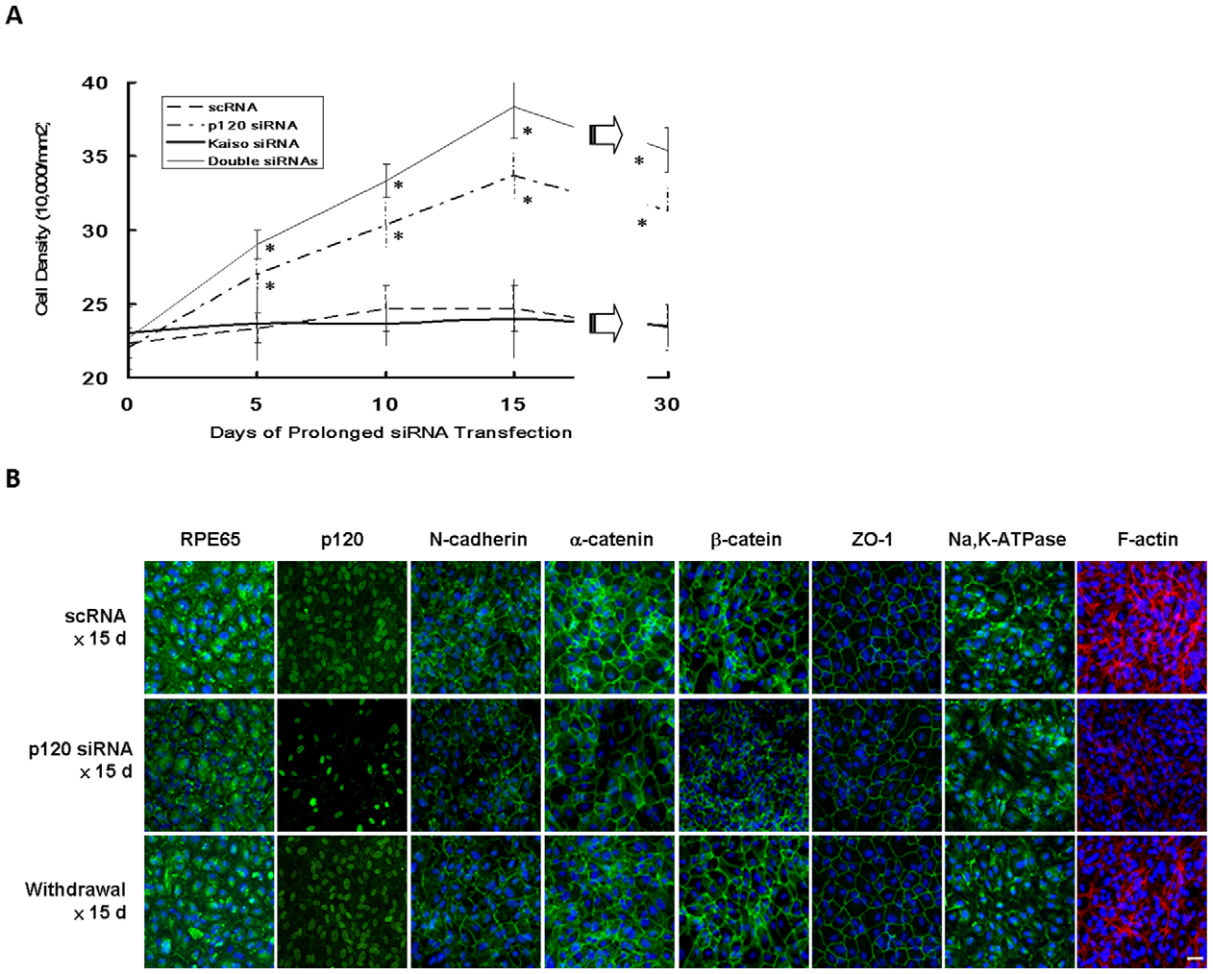
Compact ARPE monolayer with a normal phenotype without EMT after prolonged p120 knockdown. ARPE-19 cells cultured to day 7 post-confluence were transfected with 40 nM scRNA or siRNA to p120, Kaiso, or both for every 5 days until day 15, followed by withdraw for another 15 days. (A) The baseline cell density was comparable before (day 0) transfection, and reached a plateau of 24.7±2.5×10^4^/mm^2^ (n = 3) and 24.0±2.7×10^4^/mm^2^ (n = 3, *P*>0.05) in scRNA and Kaiso siRNA, respectively. In contrast, it increased to 33.7±1.5×10^4^/mm^2^ (n = 3, *P*<0.05) and 38.3±2.1×10^4^/mm^2^ (n = 3, *P*<0.01), respectively, in p120 siRNA or both siRNAs on day 15. After withdrawal, the final density of all groups slightly decreased on day 30, when same cell morphology was noted as day 15 but dome-shaped areas disappeared (* *P*<0.05). (B) Immunostaining revealed decreased peri-membranous staining of F-actin and mildly decreased junctional staining of p120, N-cadherin, α-catenin, β-catenin, ZO-1, membranous and junctional staining of RPE65, membranous staining of Na,K-ATPase after p120 siRNA transfection for 15 days. Normal expression patterns were regained after withdrawal for 15 days. Scale bar indicates 100 μm.

The above experiments based on a short pulse of 100 nM p120 siRNA knockdown for 48 h was capable of unlocking the ARPE mitotic block without inducing EMT by selective activation of p120/Kaiso and RhoA-ROCK signaling (Fig. 2–5). To determine whether proliferation could be sustained without EMT by prolonging p120 siRNA transfection, ARPE-19 cells cultured to day 7 post-confluence were transfected with 40 nM of scRNA, p120 siRNA, Kaiso siRNA or p120 and Kaiso siRNAs every 5 days for 15 days and then withdrawn for another 15 days. The starting cell density was 22.3±1.5×10^4^/mm^2^ (scRNA) 22.0±1.0×10^4^/mm^2^ (p120 siRNA), 23.0±1.0×10^4^/mm^2^ (Kaiso siRNA), and 22.7±2.1×10^4^/mm^2^ (both siRNAs), respectively, on day 7 before siRNA transfection, which was designated as day 0 of transfection. The ARPE monolayers transfected with scRNA or Kaiso siRNA virtually remained quiescent during this period in terms of morphology (not shown) or cell density (Fig. 6A). In contrast, ARPE monolayers transfected with p120 siRNA or double siRNAs continued to increase cell density to 1.5- and 1.7-fold during the entire 15 days of transfection (Fig. 6A, n = 3, *P*<0.05). After termination of siRNA transfection for another 15 days, the cell density on day 30 remained in the range of 31.3±1.6×10^4^/mm^2^ (p120 siRNA) and 35.4±1.5×10^4^/mm^2^ (both siRNAs), which was significantly higher than 23.3±1.5×10^4^/mm^2^ (scRNA) and 23.5±1.4×10^4^/mm^2^ (Kaiso siRNA) (Fig. 6A, *P*<0.05 and *P*<0.01 respectively).

Additionally, immunostaining confirmed that normal RPE phenotype, judged by membranous and cytoplasmic staining of RPE65, junctional staining of N-cadherin, α-catenin, β-catenin, ZO-1, and membranous staining of Na,K-ATPase as well as by peri-membranous staining of F-actin, was maintained in the control transfected with scRNA (Fig. 6B). Prolonged p120 siRNA knockdown for 15 days resulted in significant reduction of p120 junctional staining and increase of p120 nuclear staining, similar to what was shown in Figure 4. Neither scRNA nor p120 siRNA resulted in enhanced or positive expression of EMT markers, i.e. S100A4, and α-SMA (not shown). However, prolonged p120 siRNA knockdown notably reduced peri-junctional staining of F-actin and mildly reduced membranous and cytoplasmic staining of RPE65, junctional staining of N-cadherin, α-catenin, β-catenin, ZO-1, and membranous staining of Na-K-ATPase (Fig. 6B). Fifteen days after withdrawal of p120 siRNA, the immunostaining pattern was completely reverted to that of the control for all these 6 markers (Fig. 6B). Taken together, these results indicated that prolonged p120 siRNA knockdown further promoted the proliferative potential and cell density of post-confluent ARPE monolayer without EMT. Following withdrawal of p120 siRNA, the normal ARPE phenotype was completely restored in 15 days.

## Discussion

To unlock the mitotic block of contact-inhibited ARPE-19 cells to induce EMT, it was necessary to disrupt the cell junctions with EGTA, a notion that has been reported in TGF-β2-added porcine RPE explants [9] and in other TGF-β1-added confluent epithelial cells [49], [50]. Nonetheless, in our model system, we noted that addition of EGF and FGF-2 without TGF-β was also sufficient and necessary to induce EMT (Fig. 1). Unlike TGF-β, which induces EMT while inhibiting proliferation, EGF+FGF-2 induced EMT while promoting proliferation. This unique action by EGTA+EGF+FGF-2 could be explained by selective activation of canonical Wnt signaling because both EMT and proliferation were completely abolished by XAV399 (manuscript submitted), a specific Wnt inhibitor [37]. Interestingly, addition of EGF +FGF-2 without TGF-β was also sufficient to elicit nuclear translocation of pSmad2/3 and ZEB1/2, suggesting that canonical Wnt signaling activated by EGTA+EGF+FGF-2 was sufficient to activate Smad/ZEB signaling, which is known to cause EMT in many other epithelial cells (reviewed by Polette et al. [51]). The role of canonical Wnt signaling in contributing to the Smad signaling has also been noted in other systems (reviewed by Massague et al. [52]), including kidney [53] and intervetebral disc [54]. Taken together, we conclude that the conventional method of expanding contact-inhibited post-confluent ARPE-19 cells based on EGTA+EGF+FGF-2 runs the risk of developing EMT.

For the first time, we discovered a novel method of unlocking such mitotic block without frank disruption of cell junctions through the use of p120 siRNA (Fig. 2). As a contrast to EGTA+EGF+FGF-2, p120 siRNA knockdown did not cause EMT in part because of the lack of activation of canonical Wnt and Smad/ZEB signaling (Fig. 3). Our study further showed that this unique method based on p120 siRNA was mediated by selective activation of the p120/Kaiso signaling (Fig. 4). p120 is known to regulate cadherin-mediated adherent junctions, to stabilize them at the membrane by regulating the dynamic regulation of the actin cytoskeleton [27], to transport cadherins to the membrane, to stabilize cadherins at the membrane [17], and most importantly, to stimulate proliferation by regulating the activity of Rho GTPases including RhoA (reviewed by Rossman et al. [55]). We noted that thrombin (5U/ml, 5 min), H_2_O_2_ (200 μM, 30 min), LPA (1 μg/mL, 30 min), and HGF (10 ng/mL, 24 h), known activators of RhoA/ROCK signaling, resulted in increased proliferation in subconfluent but not post-confluent contact-inhibited ARPE-19 cells (not shown). Because p120 acts as a guanine nucleotide dissociation inhibitor by binding to and preventing RhoA activity [26], [54], we thus believe that knockdown of p120 is a unique mechanism to activate RhoA/ROCK in contact-inhibited RPE cells by releasing such an inhibition of RhoA activity but not by direct activation of RhoA/ROCK. A same result was noted in primary post-confluent hutman corneal endothelial cells [56]. Unlike our finding, others reported that p120 siRNA knockdown decreases the level of β-catenin [43] and E-cadherin in tumor epithelial cells [43], [57], VE-cadherin in vascular endothelial cells [43], [58], [59], N-cadherin in cardiac myocytes [43], [60], and vascular endothelial cells [59] without demonstrating the effect of promoting proliferation. We attributed such a disparity to their use of EDTA/trypsin dissociated but not post-confluent contact-inhibited cells because we also noted that p120 knockdown actually reduced cell proliferation when ARPE-19 cells are divided into single cells by EDTA/trypsin (unpublished observation). These data highlight a unique role of p120 in governing contact inhibition in post-confluent cultures.

The finding that p120 siRNA knockdown activates p120/Kaiso signaling was supported by a dramatic shift of its cytolocalization from the junction/membrane to the nucleus, where it was correlated with the release of nuclear Kaiso (Fig. 4B). This unique mode of action clearly involves nucleocytoplasmic shuttling of p120, which can be modulated by extrinsic factors such as cadherin binding [41], [42] and interactions with the microtubule network [42], [45]. Consistent with what has been shown in a number of cell lines [42], the extent of nuclear p120 level elicited by p120 knockdown could further be augmented by nocodazole, a microtube-disrupting agent, but diminished by taxol, a microtube-stabilizing agent (Fig. 4), implying that indeed p120 shuttling from the cytoplasm to the nucleus could further be facilitated by perturbing the microtubular network. It is worth noting that p120 nuclear translocation could not be elicited by Kaiso siRNA knockdown alone (Fig. 4B, C), which like p120 siRNA also downregulated Kaiso mRNA and protein, a finding that was also noted in porcine pulmonary artery endothelial cells [24]. The finding that Kaiso siRNA alone could not alter the nuclear level of Kaiso suggested that the homeostasis of nuclear Kaiso is tightly regulated and cannot be influenced by its own transcription and translation in a short term. In contrast, Kaiso siRNA could do so only in conjunction with p120 siRNA (Fig. 4B, C), resembling what has been shown in *H. pylori* mediated up-regulation of MMP-7 in MKN28 cells [61]. The finding that the nuclear level of Kaiso could only be reduced by p120 nuclear translocation also highlighted the specificity of p120/Kaiso signaling, and was supported by the finding that the binding domain of Kaiso with p120 is in the same domain where it binds with DNA [23]. Consequently, we observed a consistent inverse relationship between the nuclear p120 level and the nuclear Kaiso level, of which the latter was strongly correlated with nuclear BrdU labeling. These results suggest that unleashing the suppressive effect by Kaiso plays a key role in unlocking the mitotic block mediated by contact inhibition in post-confluent ARPE-19 cells.

The notion that the p120/Kaiso signaling activated by p120 siRNA was facilitated by destabilization of microtubules is supported by the finding that p120 is capable of regulating microtubule dynamics in a cadherin-independent manner [62]. Consistent with the known action of p120 in downregulating RhoA [26], [27], p120 knockdown activated RhoA-ROCK signaling (Fig. 5), which was associated with destabilization of microtubules. We discovered that p120 nuclear translocation induced by p120 knockdown was dependent on activation of RhoA-ROCK signaling and destabilization of microtubules because inhibition of RhoA by CT-4 or ROCK by Y-27632 or stabilization of microtubules by Taxol abolished p120 nuclear translocation and BrdU labeling (Fig. 5).

It has been reported that stable and continuous knockdown of p120 induces anchorage-independent growth in NIH3T3 cells [44]. Herein, we demonstrated that transient, or even prolonged (up to 15 days) exposure to p120 siRNA, followed by withdrawal could switch on and off cellular proliferation in contract-inhibited ARPE-19 cells while maintaining the expression pattern of several markers (Fig. 6). Further studies are needed to confirm that such a phenotype is consistent with their *in vivo* function, e.g., phagocytosis of photoreceptor outer segments or processing of visual cycle molecules. Furthermore, if p120 siRNA might be safely delivered *in vivo*, one may consider using it to treat diseases characterized by RPE dysfunction or degeneration due to aging and injury, where proliferation is absent or when stimulated might lead to EMT. Before such applications can be realized, the discovery made in this study should allow us to engineer functional RPE tissue *ex vivo* without the use of enzymatically-dissociated single cells for RPE transplantation with or without gene targeting.

## Materials and Methods

### Ethics Statement

No human participants were involved or animal work was conducted in this study.

### Antibodies and Reagents

Dulbecco's modified Eagle's medium (DMEM), Ham's/F12 medium, human epidermal growth factor (EGF), HEPES buffer, phosphate-buffered saline (PBS), amphotericin B, gentamicin, fetal bovine serum (FBS), and Alexa fluor-conjugated secondary antibodies IgG were purchased from Invitrogen (Carlsbad, CA). FGF-2, TGF-β1, bovine serum albumin, agarose, PCR marker, paraformaldehyde, methanol, Triton X-100, XAV939, and Hoechst 33342 dye, nocodazole, and taxol were purchased from Sigma (St. Louis, MO) or Calbiochem (La Jolla, CA). Specific monoclonal antibodies (against α-tubulin, α-SMA, β-catenin, BrdU, Kaiso, N-cadherin, Na,K-ATPase, p-Smad2/3, RPE65, and RhoA) and polyclonal antibodies (against α-catenin, connexin-43, histone, p120-catenin, S100A4, ZEB1, ZEB2, and ZO-1) were purchased from Abcam (La Jolla, CA), BD Biosciences (San Jose, CA), Cell Signaling Technology (Danvers, MA), Chemicon (Temecula, CA), Upstate (Billerica, MA), Cytoskeleton (Denvor, CO), Santa Cruz Biotechnology (Santa Cruz, CA), Sigma, and Zymed (Carlsbad, CA) (For more details see Table S1). RNeasy Mini Kit was purchased from Qiagen (Valencia, CA). High Capacity Reverse Transcription Kit and TaqMan Universal PCR Mater Mix were obtained from Applied Biosystems (Foster City, CA). Control scramble RNA (scRNA) and HP validated siRNAs to N-cadherin, α-catenin, β-catenin, ZO-1, and Kaiso were purchased from Qiagen (Catalog Number SI02663927, S102654673, SI02662478, SI02655149, and S104165924 respectively). HiPerfect^®^ siRNA and SuperFect^®^ plasmid transfection reagents were obtained from Qiagen. p120 siRNA3 was designed by ourselves and obtained from Invitrogen with the target sequence of 5′CAGAGGTGATCGCCATGCTTGGATT3′. The TCF/LEF reporter plasmid kit was from SABiosciences (Valencia, CA).

### Culture, Treatments, and Transfection

All experiments were performed using ARPE-19 (ATCC, Manassas, Virginia), passages 1–6, cultured in HEPES-buffered DMEM and Ham's F-12 (1∶1) supplemented with 10% FBS, 2 mM L-glutamine, 50 μg/ml gentamicin, and 1.25 μg/ml amphotericin B at 37°C in humidified air with 5% CO_2_. Medium was changed every 2–3 days. Upon 100% confluence, cells were continuously cultured for 7 days before being treated with 1 mM EGTA, 10 ng/ml EGF, 20 ng/ml FGF-2, or XAV939 (a specific tankyrase I inhibitor antagonist of Wnt signaling [37]) for 1 day or transfected with siRNAs (described below) for 2 days. Serial experiments were performed to confirm that the transfection efficiency of various siRNAs was approximately 90–95%. For short-pulse siRNA knockdown, transfection was performed by mixing HiPerfect^®^ siRNA transfection reagents (final dilution, 1∶300) and serum- and antibiotic-free medium to a polystyrene tube followed by incubation for 15 min at room temperature. Scrambled RNA (scRNA), or siRNA (final concentration, 100 nM) to p120, N-cadherin, α-catenin, β-catenin, ZO-1, or Kaiso was added to the above mixture, mixed gently by pipetting, and incubated for another 15 min. The transfection mixture was added dropwise to a well of a 24-well dish with ARPE cultured in 250 μl of fresh serum-containing medium. Some cultures were treated with 5 ng/ml nocodazole or 10 μM taxol in the medium during the entire period of p120 siRNA transfection. For prolonged p120 siRNA knockdown, post-confluent ARPE cells on day 7 were transfected with 40 nM scRNA or p120 siRNA added in the same manner as above every 5 days for 15 days before switch to siRNA-free fresh medium for another 15 days. The proliferative status was assessed by addition of BrdU to a final concentration of 10 μM in the culture medium for 4 h before termination. The resultant cell density per mm^2^ was determined by counting cells at 3 random fields taken by DIC microscopy at a high magnification.

### Immunofluorescence Confocal Microscopy

ARPE monolayer cultures were air-dried and fixed in 4% paraformaldehyde, pH 7.0, for 15 min at room temperature, rehydrated in PBS, incubated with 0.2% Triton X-100 for 15 min, and rinsed three times with PBS for 5 min each. After incubation with 2% BSA to block nonspecific staining for 30 min, they were incubated with appropriate primary antibodies, of which dilutions are provided in Table S1, for 16 h at 4°C. After three washes with PBS, they were incubated with corresponding Alexa Fluor-conjugated secondary IgG for 60 min. For BrdU staining and nuclear localization staining of p120, samples were fixed with 75% methanol plus 25% acetic acid for 15 min, denatured by 2 M HCl for 30 min at 37°C and neutralized by 0.1 M borate buffer, pH 8.5 for 5 min three times. Secondary antibodies IgG (donkey anti-mouse, donkey anti-rabbit, and donkey anti-goat), conjugated to Alexa 488, 555, or 633 respectively, were used at 1.7 μg/ml. The samples were then counterstained with Hoechst 33342 and analyzed with Zeiss LSM 700 confocal microscope (Thornhood, NY). Corresponding mouse and rabbit sera were used as negative controls for primary monoclonal and polyclonal antibodies, respectively.

### Preparation of Cell Lysates

To prepare protein extracts from the membrane, the cytosol, and the nucleus, we followed Qproteome Cell Compartment protocol (Qiagen). Briefly, cells were first added with Extraction Buffer CE1, which selectively disrupts, but without solubilizing, the plasma membrane, resulting in isolation of cytosolic proteins, followed by centrifugation at 100×g for 10 min to pellet plasma membranes and compartmentalized organelles, such as nuclei, mitochondria, and the endoplasmic reticulum. The pellet was then resuspended in Extraction Buffer CE2, which solubilizes the plasma membrane as well as all organelle membranes except the nuclear membrane, followed by centrifugation at 6000×g for 10 min to pellet nuclei. The supernatant contains membrane proteins and proteins from the endoplasmic reticulum and mitochondria. Finally, the pellet containing nuclei was solubilized using Extraction Buffer CE3, in which all soluble and most membrane-bound nuclear proteins are extracted, and then pelleted by centrifugation at 6800×g for 10 min.

### RhoA Activity Assay

The assay of Rho activation was performed in 100 μg of protein of cell lysates using RhoA Activation Assay Biochem Kit (Cytoskeleton) to pull down the GTP-bound form of RhoA by a GST fusion protein containing rhotekin (7–89 residues) and RBD protein using brightly colored glutathione affinity beads. The amount of activated RhoA pulled down was quantitatively determined by Western blot using anti-RhoA antibody.

### Western Blotting

The protein extracts from the above three compartments were electrophoresized on 4–15% (w/v) gradient acrylamide ready gels (Bio-Rad, Hercules, CA) under denaturing and reducing conditions, and transferred to a nitrocellulose membrane (Bio-Rad), which was then blocked with 5% (w/v) fat-free milk powder in Tris-buffered saline [10 mM Tris•HCl (pH 7.4) and 150 mM NaCl (TBS)] +0.05% Tween 20 (TBST) for 1 hour, washed three times for 10 min with TBST. After incubation with specific primary antibodies against β-catenin, LEF1, p-Smad2/3, ZEB1, and ZEB2, washed three times for 10 min with TBST, the membranes were incubated for 1 hour with horseradish peroxidase-conjugated secondary antibodies diluted in TBST using Cx43, α-tubulin, and histone as loading controls for the membranous, cytosolic, and nuclear compartments, respectively. Then membranes were washed twice for 10 min with TBST and once for 5 min with TBS, and then immunoreactive proteins were detected with Western Lighting^TM^ Chemiluminesence Reagent (PerkinElmer, Waltham, MA). Densitometry analysis was performed with computer software (ImageJ; National Institutes of Health, Bethesda, MD).

### RNA Extraction, Reverse Trascription, and Real-Time PCR

Total RNAs were extracted using RNeasy Mini Kit and reverse-transcribed using High Capacity Reverse Transcription Kit. cDNA of each cell junction component was amplified by real-time PCR (qPCR) using specific primer-probe mixtures and DNA polymerase in 7000 Real-time PCR System (Applied Biosystems, Foster City, CA). qPCR profile consisted of 10 min of initial activation at 95°C followed by 40 cycles of 15 sec denaturation at 95°C, and 1 min annealing and extension at 60°C. The genuine identity of each PCR product was confirmed by the size determination using 2% agarose gels followed by ethidium bromide staining together with PCR marker according to EC3 Imaging System (BioImaging System, Upland, CA).

### TCF/LEF Promoter Assay

Post-confluent ARPE monolayers in 24-well dishes were cotransfected with 0.4% (w/v) of the TCF/LEF construct that harbors TCF/LEF-binding sites and 0.01% (w/v) of pRL-TK internal control plasmids with 1% (w/v) SuperFect^®^ plasmid transfection reagent in the aforementioned medium. After transfection for 24 h, before adding various treatments in the fresh medium, cell lysates were assayed for firefly luciferase and Renilla luciferase activities using a Dual-Luciferase Reporter Assay System (Promega, Madison, WI) and TD-20/20 luminometer (Turner BioSystems, Sunnyvale, CA). The ratio of firefly luciferase and Renilla luciferase activities was used to determine whether the promoters are activated.

### Statistics

All data are presented as blots or images from at least three similar experiments or as mean ± S.D. for the number of experiments (n) indicated. Statistical significance was determined by one-way ANOVA and Student's unpaired *t*-test using SPSS software version 13.0 (SPSS Inc. Chicago, Illinois), where *P*<0.05 was considered statistically significant.

## Supporting Information

Figure S1 Proliferation assessed by BrdU labeling was still positive on day 4 post-confluence, but became abruptly negative from day 7 post-confluence (* ***P*** **<0.05).**
(TIF)

Figure S2 **Knockdown efficiency of siRNA to junctional proteins and Kaiso.** ARPE-19 cells cultured to post-confluence day 7 were transfected with 100 nM of scRNA or siRNA to respective junctional components (p120, N-cadherin, α-catenin, β-catenin, and ZO-1) and Kaiso for 2 days, and processed for total RNA and cell lysates. (A) qRT-PCR analysis showed that all siRNAs significantly down-regulated respective mRNA levels (n = 3, * *P*<0.05). (B) Western blot analysis confirmed that all proteins were also significantly down-regulated using β-actin as the loading control (n = 3, * *P*<0.05).
(TIF)

Figure S3 **Nuclear p120 staining by different fixatives.** ARPE-19 cells cultured to 25% confluence or day 7 post-confluence were subjected to fixation in either methanol/acetic acid (3∶1 in v/v) or 4% paraformaldehyde (w/v). Nuclear p120 staining was clearly detected in cells fixed in methanol/acetic acid but not in paraformadehyde. Scale bar indicates 100 μm.
(TIF)

Table S1 **Primary antibodies used for immunfluorescence confocal microscopy and Western blotting.**
(DOC)
