# Supplementary figures and images for: Selective Activation of p120ctn-Kaiso Signaling to Unlock Contact Inhibition of ARPE-19 Cells without Epithelial-Mesenchymal Transition
Source: PLoS One. 2012 May 9;7(5):e36864. doi: 10.1371/journal.pone.0036864 (PMC3348893; doi:10.1371/journal.pone.0036864)

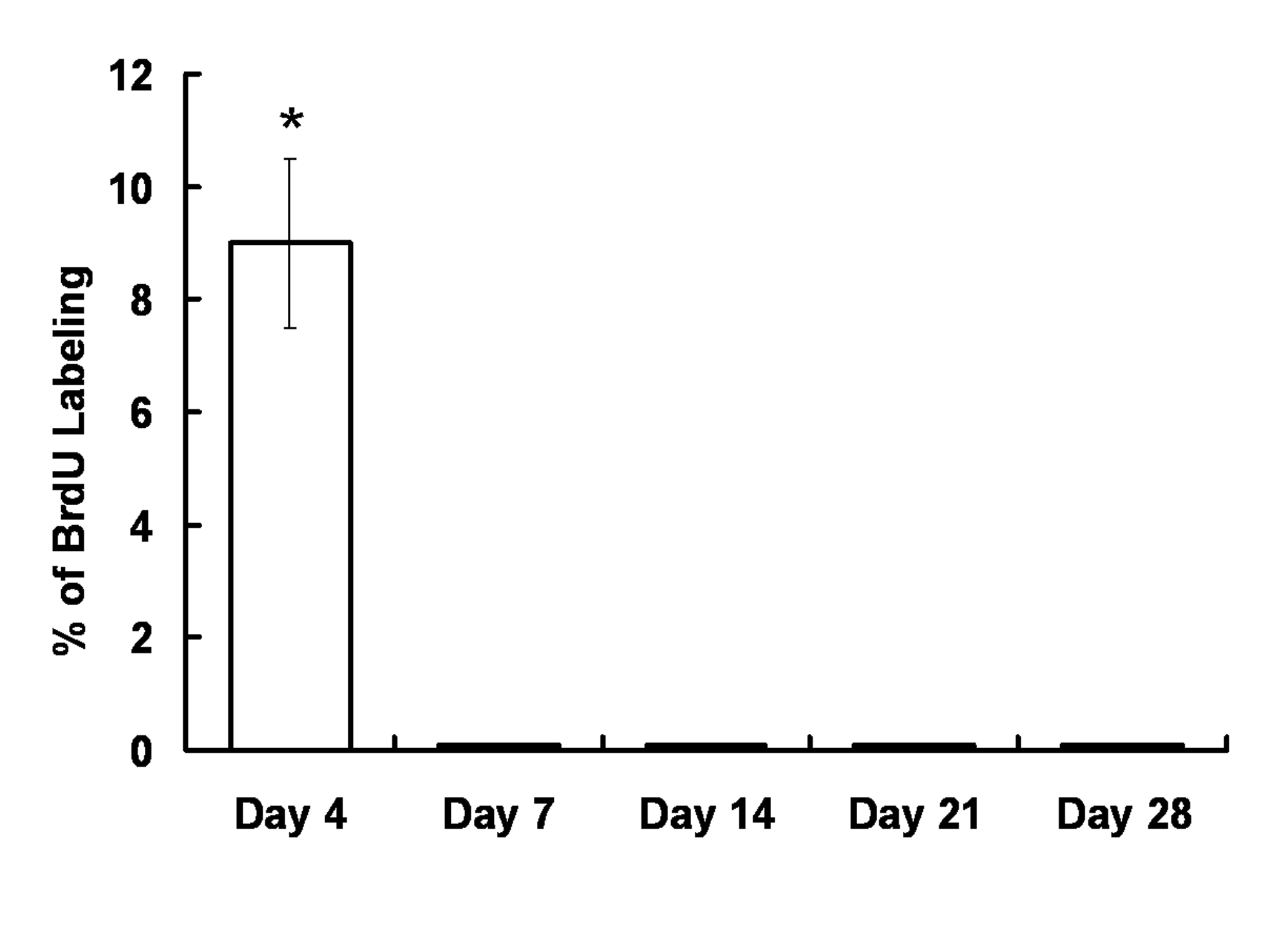

Supplement: Figure S1 — Proliferation assessed by BrdU labeling was still positive on day 4 post-confluence, but became abruptly negative from day 7 post-confluence (* P <0.05). (TIF) [file pone.0036864.s001.tif]

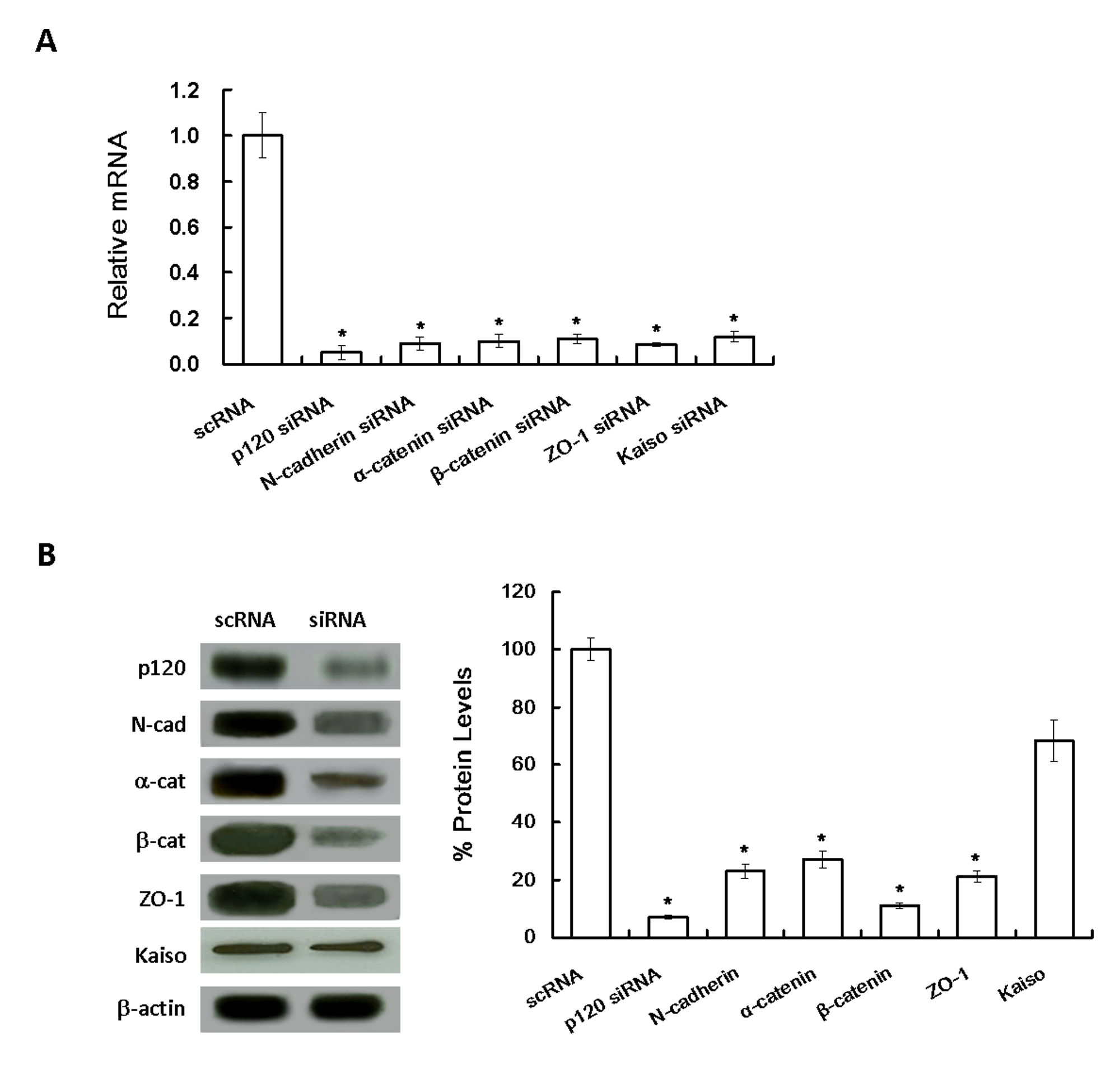

Supplement: Figure S2 — Knockdown efficiency of siRNA to junctional proteins and Kaiso. ARPE-19 cells cultured to post-confluence day 7 were transfected with 100 nM of scRNA or siRNA to respective junctional components (p120, N-cadherin, α-catenin, β-catenin, and ZO-1) and Kaiso for 2 days, and processed for total RNA and cell lysates. (A) qRT-PCR analysis showed that all siRNAs significantly down-regulated respective mRNA levels (n = 3, * P<0.05). (B) Western blot analysis confirmed that all proteins were also significantly down-regulated using β-actin as the loading control (n = 3, * P<0.05). (TIF) [file pone.0036864.s002.tif]

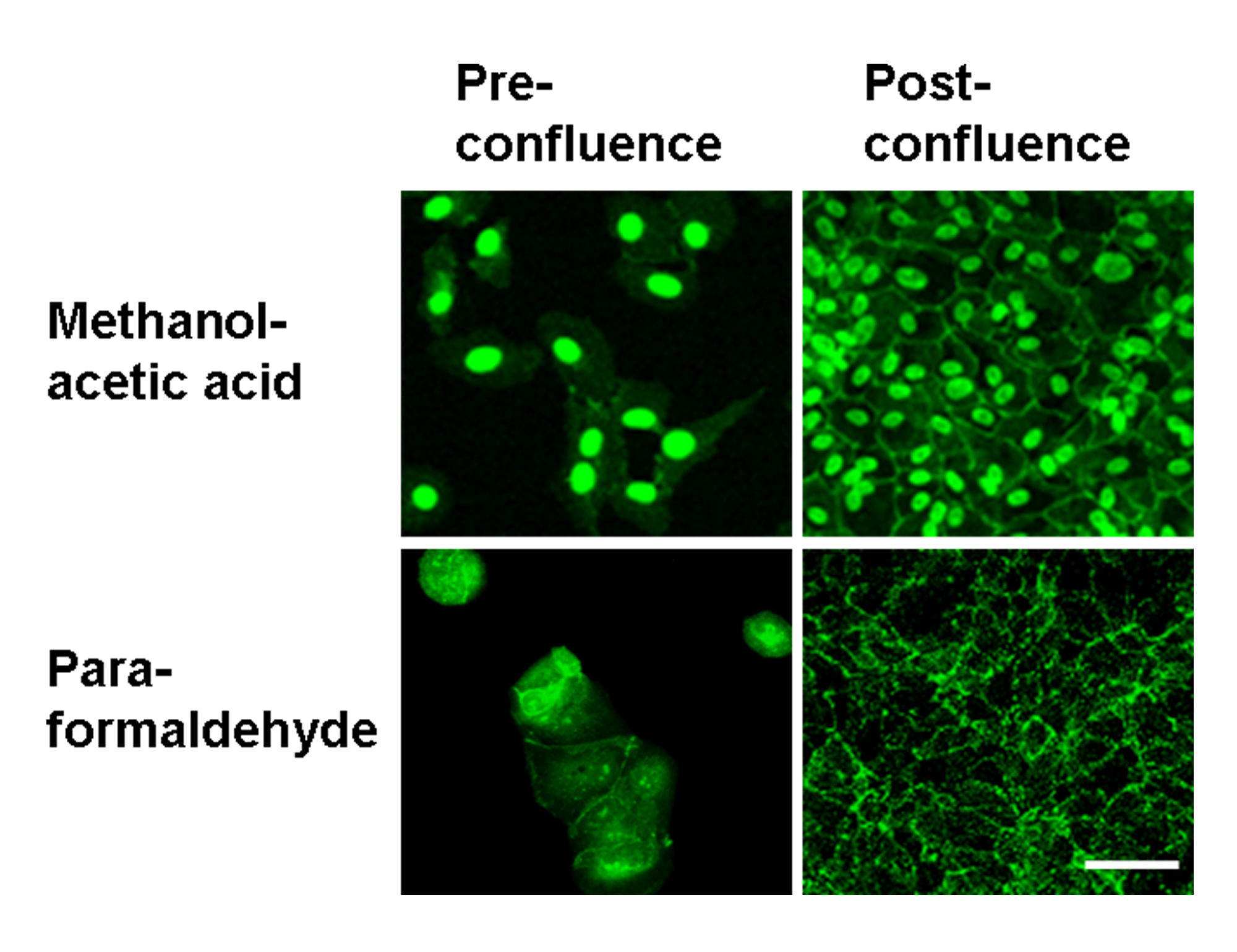

Supplement: Figure S3 — Nuclear p120 staining by different fixatives. ARPE-19 cells cultured to 25% confluence or day 7 post-confluence were subjected to fixation in either methanol/acetic acid (3∶1 in v/v) or 4% paraformaldehyde (w/v). Nuclear p120 staining was clearly detected in cells fixed in methanol/acetic acid but not in paraformadehyde. Scale bar indicates 100 μm. (TIF) [file pone.0036864.s003.tif]
