# Supplementary material for: Selective Activation of p120ctn-Kaiso Signaling to Unlock Contact Inhibition of ARPE-19 Cells without Epithelial-Mesenchymal Transition
Source: PLoS One. 2012 May 9;7(5):e36864. doi: 10.1371/journal.pone.0036864 (PMC3348893; doi:10.1371/journal.pone.0036864)
Supplement: Table S1 — Primary antibodies used for immunfluorescence confocal microscopy and Western blotting. (DOC) [file pone.0036864.s004.doc]

**Table S1. Primary antibodies used for immunfluorescence confocal microscopy and Western blotting.**

| **Antibody** | **Company** | **Cat. Number** | **Source** | **Dilution (IF:WB)** |
| --- | --- | --- | --- | --- |
| α-catenin | Sigma | C2081 | RB-PC | 1:200/1:1000 |
| α-tublin | Sigma | T9026 | MS-MC | NA/1:1000 |
| α-SMA | Abcam | ab694 | MS-MC | 1:200/NA |
| β-catenin | BD Biosciences | 610154 | MS-MC | 1:200/1:1000 |
| BrdU | Chemicon | MAB3222 | MS-MC | 1:50/NA |
| Connexin 43 | Zymed | 71-0700 | RB-PC | NA/1:1000 |
| Histone | Cell Signaling | 9715 | RB-PC | NA/1:1000 |
| Kaiso | Abcam | ab12723 | MS-MC | 1:200/1:1000 |
| LEF1 | Abcam | ab53293 | RB-MC | 1:200/1:1000 |
| N-cadherin | Abcam | ab19348 | MS-MC | 1:200/1:1000 |
| Na,K-ATPase | Upstate | 05-369 | MS-MC | 1:200/1:1000 |
| p120-catenin | Santa Cruz | sc-1101 | RB-PC | 1:50/1:500 |
| p-Smad2/3 | Cell Signaling | 9510S | RB-MC | 1:200/1:1000 |
| RhoA | Cytoskeleton | ARH03 | MS-MC | NA/1:500 |
| RPE65 | Abcam | ab78036 | MS-MC | 1:200/NA |
| S100A4 | Abcam | ab27957 | RB-PC | 1:200/NA |
| ZEB1 | Santa Cruz | sc-25388 | RB-PC | 1:50/1:500 |
| ZEB2 (SIP1) | Santa Cruz | sc-48789 | RB-PC | 1:50/1:500 |
| ZO-1 | Zymed | 61-7300 | RB-PC | 1:200/1:1000 |

IF: immunofluorescence confocal microscopy

MC: monoclonal

MS: mouse

PC: polyclonal

RB: rabbit

WB: Western blotting
